# Recurrent Distant Metastatic Endometrial Cancer Treated with Immunotherapy with Pembrolizumab: A Case Report and Literature Review

**DOI:** 10.3390/diagnostics16111703

**Published:** 2026-06-01

**Authors:** Oi-Yin Leung, Chiu-Hsuan Cheng, Dah-Ching Ding

**Affiliations:** 1 Department of Obstetrics and Gynecology, Hualien Tzu Chi Hospital, Buddhist Tzu Chi Medical Foundation, Tzu Chi University, Hualien 97004, Taiwan; roseoiyinleung@gmail.com; 2 Department of Pathology, Hualien Tzu Chi Hospital, Buddhist Tzu Chi Medical Foundation, Tzu Chi University, Hualien 97004, Taiwan; chiuhsuan.cheng@gmail.com; 3 Institute of Medical Sciences, Tzu Chi University, Hualien 97004, Taiwan

**Keywords:** endometrioid, endometrial cancer, distant metastasis, immunotherapy, pembrolizumab, MMRd

## Abstract

**Background and Clinical Significance:** Advanced-stage and recurrent metastatic endometrial cancer (EC) is a complex and challenging disease with a poor prognosis. Immunotherapy is a promising treatment for advanced and recurrent mismatch repair deficiency (MMRd) EC. **Case Presentation:** A 57-year-old female patient with stage 2 dedifferentiated EC with MMRd (immunohistochemistry revealed PMS2 loss) and stage 1 renal clear cell carcinoma received neoadjuvant chemotherapy, underwent radical hysterectomy, received adjuvant chemotherapy and radiotherapy, and underwent partial nephrectomy. Disease progression with recurrent metastases to the third rib and T12 + L1 vertebrae was observed by positron emission tomography–computed tomography (PET-CT) in April 2024. She also had concurrent papillary thyroid carcinoma. Genetic testing confirmed sensitivity to dostarlimab-gxly and pembrolizumab, leading to the initiation of pembrolizumab (200 mg Q3W) and lenvatinib (20 mg QD) in June 2024 after spine surgery. Treatment-related skin toxicities prompted a dose reduction to pembrolizumab 100 mg and lenvatinib 10 mg, but persistent discomfort led to lenvatinib discontinuation in December 2024, with symptom improvement. PET-CT in October 2024 revealed significant improvement in metastatic disease, with probable residual malignancy in the left third rib and posterior pleura, whereas recent follow-up PET-CTs in April and November 2025 showed significantly decreased ^18^F-fluorodeoxyglucose avidity in the spine and ribs compared with prior studies. She was admitted for her 30th Keytruda cycle in February 2026, with stable vital signs, normal tumor markers, and no post-infusion adverse reactions. **Conclusions:** We present a 57-year-old female patient initially diagnosed with FIGO Stage 2 EC, who subsequently developed distant metastases and was restaged as FIGO Stage 4B recurrent disease. The management of this patient illustrates the multimodal treatment approach and the critical role of molecular subtyping in guiding immunotherapeutic strategies for recurrent advanced EC.

## 1. Introduction

### 1.1. Overview of Recurrent and Metastatic Endometrial Cancer (Definition, Prognosis, and Challenges)

Advanced-stage and recurrent metastatic endometrial cancer (EC) is a complex and challenging disease with poor prognosis. It encompasses diverse presentations, including intra- and extra-abdominal metastases [1]. The 2023 International Federation of Gynecology and Obstetrics (FIGO) staging criteria have improved risk stratification for these patients, with 58% of cases changing stage upon reclassification [2]. Management typically involves cytoreductive surgery when feasible, and the degree of cytoreduction significantly affects survival outcomes [2]. Neoadjuvant chemotherapy is increasingly being used for unresectable cases [1]. Adjuvant therapy combined with radiotherapy and chemotherapy may improve survival, although there is no consensus [1]. Recent advances in molecular classification have refined prognostic risk groups and opened new treatment perspectives, including targeted immunotherapy and hormonal therapies [3]. In particular, immunotherapy is promising for the treatment of advanced and recurrent EC [4].

### 1.2. Role of Immunotherapy in Treating Advanced and Recurrent Endometrial Cancer

Immunotherapy is a promising treatment for advanced and recurrent EC, particularly in patients with mismatch repair deficiency/microsatellite instability-high (MMRd/MSI-H) [5,6]. Combining immunotherapy with chemotherapy as a first-line treatment significantly improves progression-free survival (PFS) and overall survival (OS) compared with chemotherapy alone [5]. For MMR-proficient tumors, combining immune checkpoint inhibitors (ICIs) with tyrosine kinase inhibitors has yielded compelling results [7]. The KEYNOTE-775 trial demonstrated that pembrolizumab plus lenvatinib led to significantly longer PFS and OS than chemotherapy, regardless of the MMR status, establishing a new standard of care as a second-line treatment [7]. Ongoing research is exploring immunotherapy in first-line settings and investigating novel combinations of ICIs and poly(ADP-ribose) polymerase (PARP) inhibitors [7,8].

### 1.3. Introduction to Pembrolizumab and Its Mechanism of Action (Programmed Cell Death Protein 1 Inhibition)

Pembrolizumab, an anti-programmed cell death protein 1 (PD-1) monoclonal antibody, has shown promising results in the treatment of advanced EC. It blocks the interaction between PD-1 and its ligands, thereby restoring T-cell-mediated antitumor immunity [9]. In the KEYNOTE-028 study, pembrolizumab demonstrated durable antitumor activity and a favorable safety profile in patients with heavily pretreated programmed death ligand 1 (PD-L1)-positive EC, with 13% achieving a partial response [10]. It has been approved as a monotherapy for MSI-H/MMRd EC after prior chemotherapy failure [11]. Ongoing trials are exploring its use in the earlier stages of treatment and in combination with other therapies [9]. Despite encouraging results, >50% of patients with MSI-H do not respond to treatment, underscoring the need for improved biomarkers and a better understanding of resistance mechanisms [9]. Lenvatinib combined with pembrolizumab significantly improves PFS and OS compared with chemotherapy alone in patients with advanced EC [12]. Future studies should focus on identifying patients who can benefit from chemotherapy-free treatment and exploring new combination therapies [11].

### 1.4. Objective of the Case Report and Literature Review

This case report and literature review aimed to explore the multimodal management of advanced-stage or recurrent EC with multi-organ metastases, emphasizing the role of immunotherapy, treatment response, and challenges in managing disease progression and therapy-related toxicities.

## 2. Case Presentation

This case report presented the case of a 57-year-old female patient, gravida 3 para 2 artificial abortion 1 (normal spontaneous delivery × 2), with a history of stage 2 endometrioid EC (T2N0M0) with recurrent metastasis to the third rib and T12 + L1 vertebrae (underwent orthopedic surgery with T10–L3 posterior instrumented posterolateral fusion [mega spine], L1 posterior vertebral column resection with a 38 mm expandable cage, and palliative decompression), papillary thyroid carcinoma (disappear after immunotherapy), and left renal clear cell carcinoma (stage 1) post-partial nephrectomy.

The patient had type 2 diabetes mellitus. She weighed 53.8 kg, was 165.9 cm tall, and had a body mass index of 19.55 kg/m^2^. Her menarche occurred at age 12 years, and she has been menopausal since the age of 52 years. Her mother had a history of diabetes mellitus, and her daughter had experienced menorrhagia.

Her gynecological symptoms began in 2022 (54 years old) with postmenopausal intermittent vaginal spotting. She presented to the local medical clinic for help, where endometrial thickening was noted. Hormone treatment was prescribed. However, gross hematuria was noted, and she came to the emergency room of our hospital, where hyperglycemia (blood sugar: 420 mg/dL, HbA1c: 12.0%) was noted. Glimet (Taiwan Biotech Co., Taoyuan, Taiwan) and Trajenta (Boehringer Ingelheim International GmbH, Ingelheim am Rhein, Germany) were prescribed for the treatment of diabetes mellitus. Abdominal ultrasound showed bilateral hydronephrosis. Simultaneously, abdominal computer tomography (CT) suspected endometrial and renal tumors (Figure 1). She was then referred to our gynecologic clinics for further evaluation. Per vaginal examination revealed a tumor extruding from the cervical os and located at the posterior lip of the cervix. Fixation of the Lt parametrium was also noted. A cervical biopsy was done, and the pathology revealed endometrial cancer. Tumor markers were normal within the limit (CA125, CA199, and CEA). At least stage 2 endometrioid adenocarcinoma of the endometrium was then diagnosed.

Due to surgical complexity (large tumor and involved parametrium), neoadjuvant chemotherapy (paclitaxel + carboplatin × 3 courses) was done first to shrink the tumor (NCCN guideline: uterine neoplasms) [13]. Magnetic resonance imaging was followed, and uterine size shrunk to 9.6 × 7.5 × 7.5 cm. She underwent debulking surgery (radical hysterectomy, bilateral salpingo-oophorectomy, bilateral pelvic lymph node dissection, and para-aortic lymph node dissection). Pathology revealed grade 3, FIGO stage 2 dedifferentiated carcinoma (endometrioid carcinoma with squamous differentiation and undifferentiated carcinoma). Lymphovascular space invasion was not observed. Immunohistochemistry showed normal MLH1, MSH2, and MSH6 expressions, but a loss of PMS2 (Figure 2). After surgery, she received adjuvant chemotherapy (paclitaxel + carboplatin × 3 courses) and vaginal brachytherapy (2100 cGy divided into seven fractions).

### 2.1. Imaging Study

A positron emission tomography–computed tomography (PET-CT) scan performed in April 2024 revealed increased ^18^F-fluorodeoxyglucose uptake in the left third rib and T12–L1 vertebrae (suggesting bone metastasis) and the left thyroid gland (undetermined nature) (Figure 3A–C). Bone and thyroid metastases were confirmed via biopsy. The patient underwent orthopedic surgery as mentioned above.

### 2.2. Immunotherapy and Follow-Up

Tumor marker assessment revealed elevations in carcinoembryonic antigen (CEA) at 4.9 ng/mL (normal < 3 ng/mL) and carbohydrate antigen 19-9 (CA19-9) at 81.7 U/mL (normal < 35 U/mL), while cancer antigen 125 (CA125) at 31.2 U/mL remained within the normal reference range (normal < 35 U/mL). Genetic testing showed a high tumor mutation burden and MSI-high, which confirmed sensitivity to dostarlimab-gxly and pembrolizumab, leading to the initiation of pembrolizumab (200 mg, every 3 weeks) and lenvatinib (20 mg PO every day) in June 2024 following spine surgery. Treatment-related side effects, including painful vesicles, peeling of the skin, and blistering of the feet, prompted a dose reduction to Keytruda (100 mg) and lenvatinib (10 mg PO QD). However, owing to persistent skin discomfort, she discontinued lenvatinib on her own in December 2024, leading to symptom improvement.

Serial FDG-PET/CT imaging demonstrated progressive reduction in both the metabolic activity and morphological burden of metastatic disease across three time points (October 2024, April 2025 Figure 3D, and November 2025). The concordance between decreased SUVmax values and CT-based morphological regression supports a genuine oncologic response, rather than immunotherapy-associated suppression of FDG uptake alone. Residual FDG-avid foci in the left third rib and left upper posterior pleura were interpreted as probable residual malignancy and will continue to be monitored on subsequent imaging.

### 2.3. Current Admission for the 30th Keytruda Cycle

The patient was admitted for the 30th Keytruda treatment session (100 mg) in February 2026. Clinical examination revealed stable vital signs and normal abdominal, neurological, cardiovascular, and respiratory assessments. Laboratory results were within acceptable ranges (absolute neutrophil count, 3569/μL; hemoglobin, 10.9 g/dL; platelet, 289k/μL). Tumor marker levels were within normal limits (CEA, 1.6; CA125, 8.7; and CA199, 4.4). After Keytruda administration, the patient did not experience nausea, vomiting, or any other adverse reactions. Given her stable condition, she was discharged with an outpatient department follow-up. The patient planned to receive 35 courses of Keytruda injection (2 years).

## 3. Literature Review and Discussion

### 3.1. Search Strategy

A systematic search used the keywords “endometrial cancer, distant metastasis, immunotherapy, pembrolizumab” from inception to 20 February 2026. Synonyms and related terms were also included to expand the scope of this study. Bibliographies of relevant reviews and included studies were also examined. Table 1 provides an overview of the search strategy used in the PubMed, Scopus, Web of Science, and Embase databases.

### 3.2. Current Treatment Landscape for Advanced-Stage or Recurrent Endometrial Cancer

#### 3.2.1. Standard Therapies (Surgery, Chemotherapy, Hormonal Therapy, Radiation)

Surgery is the primary and most common treatment for EC. The standard procedure is hysterectomy—the resection of the uterus—often accompanied by the resection of the fallopian tubes and ovaries (bilateral salpingo-oophorectomy) [14,15]. Lymph nodes in the pelvic area may also be resected to check for the spread [16]. In most early-stage cases, surgery alone can be curative. It provides critical information regarding cancer stage and grade, which guides further treatment decisions [16]. Recent developments include minimally invasive approaches, such as sentinel node biopsy [14].

Radiation therapy and chemotherapy are typically used after surgery to reduce the risk of recurrence or as primary treatments when surgery is not possible [17]. Radiation for EC may be delivered as external beam radiation therapy, targeting the pelvic region, or as vaginal brachytherapy, which places a radioactive source directly inside the vagina to treat the vaginal cuff [18]. Chemotherapy, commonly using combinations of drugs, such as carboplatin and paclitaxel, is preferred for advanced or high-grade ECs that have spread beyond the uterus, as it systemically targets cancer cells throughout the body [19].

Hormonal therapy is particularly relevant in EC because many tumors are hormone receptor-positive, indicating that they are driven by estrogen and progesterone [20]. Treatment typically involves progestins (such as medroxyprogesterone acetate), which counteract the stimulatory effect of estrogen on cancer cells [21]. It is most often used for low-grade, early-stage disease in women who wish to preserve fertility or for recurrent and metastatic cases where surgery is not suitable [21]. Although generally well tolerated, side effects may include weight gain, fluid retention, and mood changes [22].

#### 3.2.2. Limitations of Traditional Treatment Approaches

Surgical limitations present significant challenges, particularly for patients who are not ideal candidates for this procedure. Many patients with EC are elderly or obese or have comorbidities, such as diabetes and hypertension, making surgery high risk or sometimes impossible [23,24,25]. Standard hysterectomy permanently eliminates fertility, which is a major concern in young women who have not yet completed childbearing [26]. Additionally, surgery carries the risk of complications, such as infection, bleeding, lymphedema, and damage to surrounding organs [27]. In advanced stages, when cancer has spread beyond the uterus, surgery alone is insufficient to achieve complete tumor clearance, limiting its curative potential [1].

The limitations of radiation and chemotherapy are largely related to their lack of selectivity and significant toxicity. Radiotherapy, although effective for localized diseases, can cause long-term side effects, including bowel and bladder dysfunction, vaginal stenosis, and fatigue, severely affecting the patient’s quality of life [28]. Although chemotherapy is used for advanced or recurrent EC, it is associated with serious adverse effects, such as peripheral neuropathy, myelosuppression, nausea, and increased infection risk [29]. Furthermore, both treatments face the critical problems of drug and radiation resistance, whereby cancer cells adapt and no longer respond to therapy, leading to treatment failure, especially in recurrent or metastatic cases [30].

The limitations of hormonal therapy are primarily related to resistance and a narrow range of applicability. Hormonal therapy is only effective for hormone receptor-positive tumors, excluding patients with high-grade or receptor-negative ECs [31]. Over time, many tumors develop resistance to progestins, rendering the therapy ineffective for long-term disease control [32]. Response rates are relatively modest, ranging from 15% to 30%, even in eligible patients [33]. Additionally, prolonged hormonal therapy carries risks, such as thromboembolic events (blood clots), weight gain, and cardiovascular complications, limiting its tolerability in patients who already have underlying metabolic conditions that are common in patients with EC [34].

### 3.3. Molecular Classification of Endometrial Cancer (EC)

The Molecular Classification of Endometrial Cancer based on The Cancer Genome Atlas and its clinically adapted version (ProMisE) are stated below [35,36].

POLE Ultramutated (POLEmut) represents a distinct and favorable subgroup of ECs characterized by somatic mutations in the exonuclease domain of the POLE gene, which is responsible for DNA proofreading during replication [37]. These mutations result in extremely high tumor mutational burden (TMB), producing thousands of mutations per tumor [38]. Despite their high-grade histological appearance, which traditionally suggests aggressive behavior, POLEmut tumors have an excellent prognosis with significantly low recurrence rates [39]. Patients in this group may benefit from de-escalation of adjuvant therapy, avoiding unnecessary radiation or chemotherapy [38]. Emerging evidence also suggests strong responsiveness to ICIs driven by their high immunogenicity [40].

MMRd/MSI-H tumors arise from defects in the MMR system, either through germline mutations (as observed in Lynch syndrome involving MLH1, MSH2, MSH6, or PMS2) or somatic MLH1 promoter hypermethylation [41]. These defects lead to the accumulation of errors in microsatellite regions of DNA, resulting in microsatellite instability [42]. Crucially, MMR status can be reliably assessed using immunohistochemistry (IHC) for the four MMR proteins (MLH1, MSH2, MSH6, and PMS2) performed on routine formalin-fixed, paraffin-embedded (FFPE) tumor tissue—a method that is widely available, technically straightforward, rapidly interpretable, and considerably more cost-efficient than molecular-based MSI testing by PCR or next-generation sequencing [43]. The loss of nuclear staining for one or more MMR proteins on IHC directly identifies MMRd tumors, providing actionable information within standard pathology workflows without requiring specialized platforms [43]. MMRd ECs have an intermediate prognosis and are clinically significant because they are strong candidates for immunotherapy, particularly PD-1/PD-L1 checkpoint inhibitors, such as pembrolizumab, which have shown remarkable response rates in this subgroup [44]. Furthermore, identifying MMRd tumors also has critical implications in screening patients and their families for Lynch syndrome [45].

The copy number low/no-specific molecular profile (NSMP) and copy number high/TP53-mutated (p53abn) represent the remaining two molecular subgroups with contrasting outcomes [46]. The NSMP group is the largest subgroup and is predominantly composed of low-grade endometrioid tumors that are hormone receptor-positive and have an intermediate-to-favorable prognosis [46]. These tumors may benefit from hormonal therapy and are currently under investigation as targeted treatment strategies [46]. In contrast, the p53abn subgroup, which includes serous carcinomas and high-grade endometrioid tumors, has the worst prognosis among the four groups and is characterized by widespread chromosomal instability, high copy number alterations, and aggressive clinical behavior [47]. These tumors are typically treated with aggressive chemotherapy regimens, and targeted therapies, such as human epidermal growth factor receptor 2 (HER2)-directed treatment, are being explored because HER2 overexpression is frequently observed in this subgroup [48].

A single-institution study confirmed these prognostic trends, with 3-year PFS rates of 97% for POLE, 80% for MSI-H/MMRd, and 46% for copy number-high (p53abn) subtypes [49]. Molecular classification is recommended for all ECs, particularly high-grade tumors, to guide treatment decisions and assess treatment efficacy in biologically similar tumors [50].

### 3.4. Role of Immunotherapy in EC

#### 3.4.1. Mechanism of Pembrolizumab in Treating Microsatellite Instability-High/Mismatch Repair Deficiency EC

Pembrolizumab is a monoclonal antibody that inhibits the PD-1 pathway and restores T-cell activity against tumors [51]. Its mechanism involves blocking the interaction between PD-1 on T cells and PD-L1 on tumor cells, thereby disrupting immune suppression in the tumor microenvironment [52]. Molecular simulations have revealed that pembrolizumab binding to PD-1 causes conformational changes, particularly in the C’D and FG loops, which prevent PD-1/PD-L1 interactions [53]. This non-cross-resistance mechanism has shown superior efficacy to ipilimumab in clinical trials of advanced melanoma and other cancers [51,54]. Factors such as PD-L1 expression and tumor mutation burden can predict response to pembrolizumab [51]. Although pembrolizumab has demonstrated broad-spectrum efficacy and safety, ongoing research is aimed at optimizing patient selection, managing immune-related adverse events (irAEs), and exploring novel strategies, such as fecal microbiota transplantation, to enhance its effectiveness [51].

#### 3.4.2. Clinical Trials and Food and Drug Administration Approval

##### KEYNOTE-158 Study

KEYNOTE-158 was a multicenter, nonrandomized, open-label, multicohort phase II basket trial evaluating the efficacy of pembrolizumab across multiple solid tumor types, including two endometrial carcinoma cohorts: Cohort D (EC regardless of MMR status) and Cohort K (any MSI-H/MMRd solid tumors) [55]. Among 90 patients with MSI-H or MMRd endometrial carcinoma, pembrolizumab achieved an objective response rate (ORR) of 46%, comprising 12% complete responses and 33% partial responses, at a median follow-up of 16.0 months [56]. Updated long-term analyses confirmed the durability of these responses, with the ORR reaching 48%; among responding patients, 88% maintained their response for at least one year, 73% for at least two years, and 68% for at least three years [57]. High tumor mutational burden (TMB ≥10 mutations per megabase) was identified as an additional favorable predictive biomarker, with an ORR of 29% in the TMB-high group compared with 6% in the non-TMB-high group [57]. Based on these results, the FDA approved pembrolizumab on 21 March 2022, as a single-agent therapy for patients with advanced MSI-H or MMRd endometrial carcinoma who experienced disease progression following prior systemic therapy and were ineligible for curative surgery or radiation [29].

##### Dostarlimab (Jemperli)—RUBY Trial

In July 2023, the FDA approved dostarlimab in combination with carboplatin and paclitaxel chemotherapy for patients with newly diagnosed primary advanced or recurrent MMRd/MSI-H endometrial carcinoma, based on results from the phase III RUBY trial [58]. The RUBY trial demonstrated a marked improvement in PFS, with a median PFS of 30.0 months in the dostarlimab plus chemotherapy arm compared with 7.7 months in the chemotherapy-alone arm among patients with MMRd/MSI-H tumors. In 2024, updated RUBY trial data supported a further expanded indication encompassing patients with primary advanced or recurrent EC regardless of MMR status, demonstrating a 72% reduction in the risk of progression or death in the MMRd/MSI-H subgroup and a 36% reduction in the overall study population [58]. An ongoing phase III trial, DOMENICA, continues to evaluate dostarlimab as first-line monotherapy versus chemotherapy in advanced and metastatic MMRd endometrial carcinoma [59].

##### Pembrolizumab (Keytruda)—KEYNOTE-868/NRG-GY018 Trial

On 17 June 2024, the FDA approved pembrolizumab in combination with carboplatin and paclitaxel, followed by pembrolizumab maintenance monotherapy, for adults with primary advanced or recurrent endometrial carcinoma, based on the KEYNOTE-868/NRG-GY018 trial [29]. This randomized phase III trial enrolled 810 patients, including 222 with MMRd tumors and 588 with pMMR tumors. In the MMRd cohort, the 24-month PFS rate was 74% in the pembrolizumab plus chemotherapy arm versus 38% in the placebo plus chemotherapy arm, representing a 70% reduction in the relative risk of progression or death. In the pMMR cohort, median PFS was 13.1 months with pembrolizumab versus 8.7 months with placebo [29]. The KEYNOTE-B21/ENGOT-en11/GOG-3053 trial showed adjuvant pembrolizumab added to chemotherapy did not improve disease-free survival in the overall high-risk endometrial cancer population following curative-intent surgery, though a significant PFS benefit was observed in the dMMR subgroup [60].

##### Durvalumab (Imfinzi)—DUO-E Trial

On 14 June 2024, the FDA approved durvalumab in combination with carboplatin and paclitaxel chemotherapy, followed by durvalumab maintenance monotherapy, for patients with MMRd primary advanced or recurrent endometrial carcinoma, based on results from the DUO-E phase III trial [61]. In the overall study population, the median PFS was 15.4 months in the durvalumab plus chemotherapy arm compared with 12.6 months in the chemotherapy-alone arm. Patients with MMRd tumors derived the greatest clinical benefit. Notably, DUO-E was the first phase III trial to incorporate PARP inhibitor maintenance in combination with immunotherapy in endometrial carcinoma, with the durvalumab plus olaparib maintenance arm demonstrating a median duration of response of 18.7 months compared with 7.6 months in the control arm, along with a 32% reduction in the risk of secondary progression or death [61]. These findings provide encouraging support for further investigation of PARP inhibitor-based combination strategies, particularly in the pMMR subgroup.

Collectively, the results of these three pivotal trials have established immunotherapy-based combination regimens as the new standard of care for patients with advanced endometrial carcinoma (Table 2). An important unresolved challenge remains optimizing the efficacy of checkpoint inhibition in patients with pMMR tumors, with ongoing research focused on identifying molecular subgroups and combination strategies most likely to confer benefit in this population.

#### 3.4.3. Effectiveness in Programmed Death Ligand 1 (PD-L1)-Positive vs. PD-L1-Negative Tumors

Immunotherapy has shown promise for EC, particularly for MSI-H/MMRd tumors. These tumors exhibit higher neoantigen loads and tumor-infiltrating lymphocytes (TILs) than microsatellite-stable (MSS) tumors [62]. PD-1 inhibitors have demonstrated response rates of 49–57% in MSI-H/MMRd-advanced EC, whereas PD-L1 inhibitors show response rates of 27–43% [63]. In MSS- or PD-L1-positive advanced EC, the response rates to PD-1/PD-L1 inhibitors range from 3 to 23% [63]. MSI is associated with increased TILs and higher PD-1/PD-L1 expression, suggesting that MSI can be a biomarker for immunotherapy response in EC [64]. Combination therapies, such as lenvatinib and pembrolizumab, have shown promise in advanced EC, which is non-MSI-H/MMRd [63]. Further studies are required to optimize the immunotherapeutic strategies for EC [65].

### 3.5. Comparison of Pembrolizumab with Other Targeted Therapies

#### 3.5.1. Combination with Lenvatinib (Tyrosine Kinase Inhibitor) for Microsatellite-Stable/pMMR Tumors

In 2019, the FDA granted breakthrough therapy designation to a combination of lenvatinib and pembrolizumab for the treatment of patients with advanced MSS EC who experienced disease progression following at least one prior systemic treatment [66]. Clinical trials have suggested that this combination demonstrates promising antitumor activity in patients with disease progression after prior therapy, irrespective of the tumor MSI status, and has a manageable toxicity profile [67]. It also resulted in a notably extended PFS among patients with advanced EC (pMMR population: 6.6 vs. 3.8 months; hazard ratio for progression or death, 0.60; 95% confidence interval [CI], 0.50 to 0.72; *p* < 0.001; overall: 7.2 vs. 3.8 months; hazard ratio, 0.56; 95% CI, 0.47 to 0.66; *p* < 0.001) and OS (pMMR population: 17.4 vs. 12.0 months; hazard ratio for death, 0.68; 95% CI, 0.56 to 0.84; *p* < 0.001; overall: 18.3 vs. 11.4 months; hazard ratio, 0.62; 95% CI, 0.51 to 0.75; *p* < 0.001) compared with chemotherapy in individuals with advanced EC [12,67]. The combination of pembrolizumab with lenvatinib showed advantages over the paclitaxel-carboplatin regimen in the postadjuvant subgroup, resulting in extended PFS and improved OS. According to the findings of Study 309/KEYNOTE-775, this combination therapy continues to be considered a standard treatment option for patients with advanced or recurrent EC that has progressed.

#### 3.5.2. Emerging Checkpoint Inhibitors (Nivolumab, Dostarlimab)

The role of checkpoint inhibitors in EC has been evolving over the years [68]. It is emerging as a management option for EC, both as a single agent and in combination with other targeted agents [66]. Ongoing studies have investigated the potential efficacy of ICIs in combination with chemotherapy, radiotherapy, and other targeted treatments [69]. For instance, patients with recurrent or metastatic EC show a persistent response to nivolumab without severe toxicity [70]. The combination of pembrolizumab with lenvatinib and dostarlimab has received approval as a second-line treatment alternative; additionally, either dostarlimab or pembrolizumab may be incorporated into first-line platinum-based chemotherapy for malignancies characterized by MMRd [68]. Although the integration of these therapies into clinical settings has resulted in enhanced overall response rates and survival outcomes, a significant number of patients continue to experience limited benefits, potentially attributable to various intrinsic and adaptive resistance mechanisms associated with immunotherapy [68].

### 3.6. Predictive Biomarkers for Immunotherapy Response

#### 3.6.1. MSI-H vs. MSS Status

The MSI-H status is a significant indicator of ICI effectiveness [71]. Tumors exhibiting high microsatellite instability (MSI-H) generally have elevated mutational burden and increased neoantigen levels [71]. They can produce more neoantigens recognized by the immune system, making them more favorable than MSS tumors for immunotherapeutic approaches [72]. One study showed that ICIs in MSI-H cancers resulted in a more favorable time to the next treatment (adjusted hazard ratio [aHR], 0.18; *p* = 0.001) and OS (aHR, 0.29; *p* = 0.045), providing evidence of their effectiveness [73]. Moreover, the anti-PD-1 dostarlimab (TSR-042) was approved as a monotherapy for patients with MSI-H EC who showed progression following platinum-based therapies [74].

#### 3.6.2. Tumor Mutational Burden

TMB also serves as a predictive biomarker, indicating a patient’s response to various immunotherapies [75]. A higher TMB is frequently associated with a greater immunotherapy response, particularly with ICIs [71]. Tumors burdened with many mutations generate more neoantigens and novel proteins resulting from mutations that are easier for the immune system to recognize [72]. In a study investigating MSI and TMB as predictive biomarkers, ICIs showed a more favorable response in MSI-H cancers, and MSI status was concordant with TMB ≥ 10 in 94.3% of cases [73]. This shows that MSI and TMB can potentially serve as strong predictors of the response to ICIs. Evaluation of TMB provides clinicians with critical insights that can guide the selection of optimal treatment strategies.

#### 3.6.3. PD-L1 Expression

The association between PD-L1 expression and the prognosis of patients with EC has been thoroughly investigated, yielding mixed results when examining its correlation with tumor or immune cells and their links to survival [76]. Some studies have indicated a positive association between PD-L1 expression and features, such as poor differentiation and advanced stages of endometrial carcinoma [77]. A 2021 cohort study found that PD-L1 positivity was more frequent in high-grade tumors than in low-grade tumors [78]. Additionally, PD-L1 expression in immune cells correlated with lymphovascular space invasion, non-endometrioid histology, and deep myometrial infiltration. Furthermore, among patients diagnosed with high-risk EC, PD-L1 positivity in tumor cells was associated with a better prognosis [77,78].

### 3.7. Future Perspectives

#### 3.7.1. Resistance Mechanisms to Pembrolizumab

Development of resistance mechanisms is a major challenge in the treatment of EC with pembrolizumab. During the regulation of tumor growth and resistance, the immune system functions in three distinct stages: elimination, equilibrium, and escape [79]. Throughout these stages, the immunogenic properties of tumors are altered, and resistance to immunotherapy develops. Tumors can exhibit intrinsic factors, such as a low mutational burden or the absence of neoantigen expression, which may limit the efficacy of ICIs. Some patients show primary resistance to anti-PD-1 therapy, adaptive resistance during initial sensitivity, or acquired resistance after relapse [80]. Although research has demonstrated that elevated PD-1/PD-L1 expression is associated with a favorable prognosis, tumors can acquire resistance to treatment by increasing PD-1/PD-L1 expression [76]. The association between PD-1/PD-L1 expression and treatment outcome is complex, highlighting the need for effective treatment strategies. Therefore, an ongoing assessment of PD-L1 expression levels throughout treatment is essential. Therefore, it is important to formulate an approach that promotes the effectiveness of immunotherapy against resistance.

#### 3.7.2. Management of Immune-Related Adverse Effects

Immunotherapy is an immensely beneficial approach for patients; however, it also has several irAEs that must be controlled with utmost care. This study suggests that PD-1 inhibitors may contribute to the development of rapid irAEs, potentially through compensatory activation of the key receptor–ligand pairs C-X-C motif chemokine receptor 4 and C-X-C motif chemokine receptor 6 [81]. Dermatological irAEs are the most prevalent toxicities associated with ICIs, typically manifesting within 3–6 weeks following the of treatment commencement [82]. Moreover, gastrointestinal toxicity induced by ICIs can affect any region of the digestive tract, from the oral mucosa to the rectum, with both the small and large intestines being predominantly impacted, and is more frequently observed among those receiving anti-cytotoxic t-lymphocyte-associated protein 4 agents (10–25% prevalence) or combination therapies (~20%) than among those treated solely with anti-PD-1 agents (1–5%) [82]. Despite the increasing use of immunotherapy, the understanding of the mechanisms underlying these toxicities and the most effective treatments remains limited. Therefore, it is crucial to gain insight into the molecular foundations of irAEs.

#### 3.7.3. Cost-Effectiveness and Accessibility of Immunotherapy

The cost of immunotherapy, including pembrolizumab, is a major barrier to patient access. There has been an increasing awareness of the financial toxicity associated with cancer treatment, particularly immunotherapy [83]. High treatment costs can limit access, especially to healthcare systems with limited budgets. A study conducted in the United States found that dostarlimab plus chemotherapy is more cost-effective than pembrolizumab plus chemotherapy in patients with advanced EC, regardless of MMR status [84]. However, compared with chemotherapy alone, dostarlimab plus chemotherapy demonstrated greater cost-effectiveness, specifically within the MMRd/MSI-H population [84].

The effectiveness of immunotherapy in the diagnosis and treatment of EC and its cost-effectiveness nexus are crucial topics that need to be assessed to inform funding decisions for developing and implementing this promising approach.

#### 3.7.4. Ongoing Clinical Trials and Novel Combination

In light of the rapid development of immunotherapy applications, further studies comparing the outcomes of immunotherapy and chemotherapy are being conducted.

Other clinical trials to evaluate the efficacy of these novel combinations are currently underway. In another clinical trial (NCT06952504), researchers conducted a phase 3 randomized, open-label, multicenter study to evaluate the efficacy and safety of sacituzumab tirumotecan (Sac-TMT, MK-2870) in combination with pembrolizumab compared with pembrolizumab alone as first-line maintenance therapy for participants diagnosed with mismatch repair-proficient EC (TroFuse-033/GOG-3119/ENGOT-en29). Sac-TMT is an antibody-drug conjugate that targets specific sites on cancer cells to deliver therapeutic agents that destroy them. This study aimed to determine whether patients receiving sac-TMT in combination with pembrolizumab have improved OS and experienced less disease progression than those treated with pembrolizumab alone.

These investigations may contribute to a better understanding of the efficacy of immunotherapeutic agents relative to other treatments and help build a clinical consensus on EC treatment choice.

#### 3.7.5. Personalized Treatment Approaches Based on Molecular Profiling

Molecular classification offers a reliable system for categorizing EC and facilitates the identification of tumors with similar biological characteristics. This approach to molecular subtyping provides prognostic and predictive insights that benefit both patients and healthcare providers, while establishing a framework for further stratification [82,85]. Implementing molecular classification of samples obtained from endometrial biopsies or curettage enables informed decision-making from the initial diagnosis onward. It is evolving to provide comprehensive insights that will inform and enhance treatment strategies for EC [73,74].

Consequently, it is essential to prioritize clinical trials targeting EC defined by molecular characteristics to assess treatment effectiveness across biologically comparable tumors and enhance patient outcomes in this area of disease [76]. In this evolving landscape, we expect greater clarity regarding each molecular subtype, which will contribute to improvements in the systemic therapies available to treat EC.

## 4. Conclusions

This case report demonstrates that accurate molecular subtyping through straightforward and cost-efficient immunohistochemical testing is not merely a research tool, but a clinically indispensable step in the management of recurrent endometrial carcinoma. The identification of PMS2 loss by IHC, a technically simple, widely available, and economically accessible assay performable on routine FFPE tissue, established the MMRd/MSI-H status of this tumor and directly guided the decision to initiate pembrolizumab-based immunotherapy. This single diagnostic result, obtainable within standard pathology workflows without the need for specialized molecular platforms, unlocked access to a therapeutic strategy that achieved remarkable and durable metastatic disease control, including near-complete resolution of multi-organ metastases and probable remission of the concurrent papillary thyroid carcinoma over 30 cycles of treatment.

The clinical course presented here reinforces several definitive lessons. First, MMR IHC should be regarded as an essential and universally accessible component of the diagnostic workup for all patients with recurrent or advanced endometrial carcinoma, irrespective of institutional resources, given its low cost, technical simplicity, and direct therapeutic implications. Second, pembrolizumab demonstrates meaningful and sustained efficacy in MMRd endometrial carcinoma with extensive metastatic burden, including bone and pulmonary involvement, supporting its use even in heavily pretreated patients with complex oncologic histories. Third, the management of treatment-related toxicities, exemplified here by lenvatinib-associated cutaneous adverse effects leading to self-discontinuation, remains a critical and underappreciated determinant of long-term treatment adherence and requires proactive multidisciplinary monitoring. Finally, this case highlights the emerging importance of synchronous triple primary malignancies as a clinical entity warranting vigilance, particularly in patients with underlying hereditary cancer predisposition, where a single IHC-based MMR assessment may carry implications extending beyond the index tumor.

In conclusion, the integration of simple, cost-efficient molecular diagnostics into routine gynecologic oncology practice has the potential to transform the treatment trajectory of patients with advanced endometrial carcinoma. Expanding the universal adoption of MMR IHC testing across all clinical settings—including resource-limited environments—is both feasible and imperative to ensure that patients receive molecularly guided, evidence-based immunotherapy in a timely manner.

## Figures and Tables

**Figure 1:**
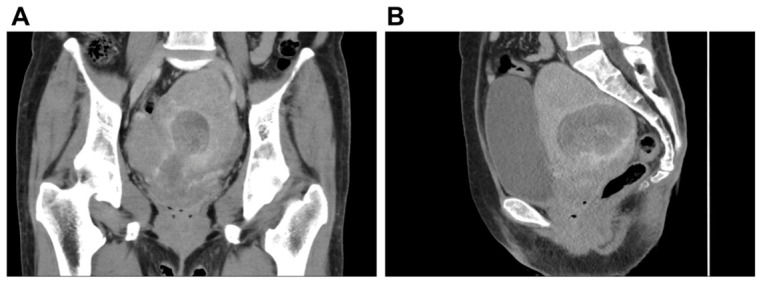
Computer tomography of the uterine tumor. (**A**) Coronal view. (**B**) Sagittal view. A hypoechoic region is represented for an endometrial tumor from the uterus to the cervix. Tumor size is measuring 10.3 × 6.4 × 4.3 cm. Uterine size is 13.8 × 9.3 × 10.3 cm.

**Figure 2:**
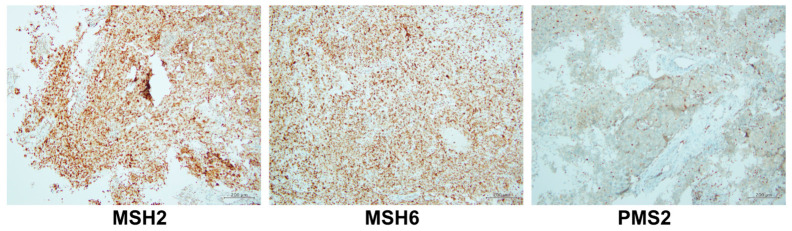
Immunohistochemistry of endometrial cancer. Positive staining for MSH2 and MSH6 and negative staining for PMS2. Scale bar = 200 μm.

**Figure 3:**
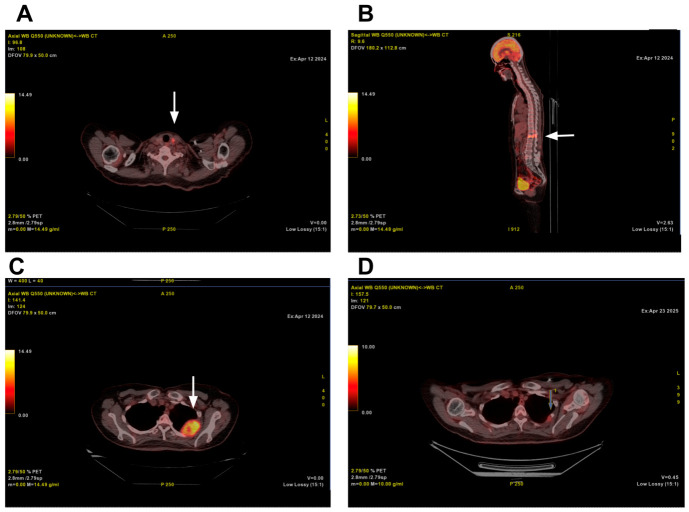
Positron emission tomography–computed tomography images demonstrating metastatic lesions. (**A**) Hypermetabolic lesion in the left thyroid gland (arrow), (**B**) T12–L1 vertebrae (arrow), and (**C**) left third rib (arrow) observed in April 2024. (**D**) Persistent metastatic lesion in the left third rib (arrow) observed in April 2025.

**Table 1:** Search strategy for the literature.

Items	Specification
Timeframe	From inception to 20 February 2026
Database	PubMed, Scopus, Web of Science, and Embase
Search terms used	“endometrial cancer, distant metastasis, immunotherapy, pembrolizumab”
Inclusion and exclusion criteria	All references were Science Citation Index-indexed articles written in English
Selection process	Two independent reviewers evaluated the titles and abstracts to determine eligibility

**Table 2:** Summary of the clinical trials.

Trial	Drug	Phase	Population	MMR Status	Key Efficacy	FDA Approval	Setting
KEYNOTE-158	Pembrolizumab	Phase II	90 pts	MMRd only	ORR 46%; CR 12%; mPFS 13.1 mo; mOS not reached; 3-yr OS 60%	1 March 2022	Previously treated (≥1 prior line)
Cohorts D & K	200 mg q3w (monotherapy)		MSI-H/MMRd EC			Single agent, previously treated, MSI-H/MMRd, nonsurgical candidates	
RUBY	Dostarlimab	Phase III	494 pts	MMRd All-comer	mPFS 30 mo (MMRd) vs. 8 mo (chemo); 72% ↓ risk of progression in MMRd; 36% ↓ in all-comer	July 2023 (MMRd)	First-line advanced or recurrent
NCT03981796	+carboplatin/paclitaxel → maintenance		Advanced/recurrent EC			August 2024 (all-comer, expanded)	
KEYNOTE-868/NRG-GY018	Pembrolizumab	Phase III	810 pts	MMRd pMMR	MMRd: PFS 74% vs. 38% (placebo); HR 0.30; pMMR: mPFS 13.1 vs. 8.7 mo; HR 0.54	17 June 2024	First-line advanced or recurrent
NCT03914612	+carboplatin/paclitaxel → maintenance		(222 MMRd; 588 pMMR)			Both MMRd and pMMR populations	
DUO-E	Durvalumab	Phase III	718 pts	MMRd pMMR	mPFS 15.4 mo (durvalumab) vs. 12.6 mo (chemo); DOR 18.7 vs. 7.6 mo; 32% ↓ risk 2nd progression	14 June 2024	First-line advanced or recurrent
NCT04498117	+carboplatin/paclitaxel → maintenance ± olaparib		Advanced/recurrent EC			MMRd population; first phase III with ICI + PARP inhibitor	

ICI, immune checkpoint inhibitor; PFS, progression-free survival; ORR, objective response rate; OS, overall survival; CR, complete response. ↓ decreased.

## Data Availability

The original contributions presented in this study are included in the article. Further inquiries can be directed to the corresponding author.

## Abbreviations

The following abbreviations are used in this manuscript:

EC	endometrial cancer
FIGO	International Federation of Gynecology and Obstetrics
MMRd	mismatch repair deficiency
MSI-H	microsatellite instability high
PARP	poly(ADP-ribose) polymerase
PD-1	programmed cell death protein 1
PD-L1	Programmed death-ligand 1
PET-CT	Positron emission tomography-computed tomography
FDG	^18^F-fluorodeoxyglucose
PO QD	per oral every day
EBRT	external beam radiation therapy
TCGA	The Cancer Genome Atlas
POLEmut	POLE ultramutated
NSMP	No Specific Molecular Profile
p53abn	TP53-mutated
TILs	tumor-infiltrating lymphocytes
MSS	microsatellite-stable
ORR	objective response rate
irAEs	immune-related adverse effects
OS	overall survival
PFS	progression-free survival
ICI	immune checkpoint inhibitor
BICR	Blinded Independent Central Review
